# Non-Cancer-Related Causes' Impact on Lung Cancer Survival: SEER Analysis

**DOI:** 10.1007/s13193-025-02458-7

**Published:** 2025-10-24

**Authors:** Lin-miao Zeng, Jie-huang Zhang, Li-ping Lin, Bi-zhen Li, Shuo Lin, Bin Song, Xiao-lian Yu

**Affiliations:** 1 Department of Respiratory and Critical Care Medicine, Mindong Hospital Affiliated to Fujian Medical University, Fu’an, Fujian, 355000 China; 2 Fujian Medical University, Fuzhou, Fujian, 350000 China; 3 Department of Medical Imaging, Mindong Hospital Affiliated to Fujian Medical University, Fu’an, Fujian, 355000 China

**Keywords:** Lung cancer, Standardized mortality ratio, Non-cancer-related cause of death, Competing risk analysis, SEER database

## Abstract

As lung cancer (LC) patients survive longer, non-cancer-related mortality risks are increasingly prominent, yet temporal trends and risk factors remain unclear. This study systematically evaluates standardized mortality ratios (SMRs) for non-cancer deaths and their temporal dynamics. We analyzed 371,177 lung cancer patients from tthe Surveillance, Epidemiology, and End Results (SEER) database (2000–2019), categorizing non-cancer-related deaths into six groups: infectious, diabetes, cardiovascular, respiratory, digestive, and others. SMRs were calculated, and Fine-Gray models assessed mortality risks. Non-cancer-related deaths rose significantly over time, increasing from 7.72% within 1 year to 53.97% beyond 5 years post-diagnosis (overall SMR = 2.24, 95% CI 2.22–2.26), surpassing cancer-specific mortality after 5 years. Cardiovascular (SMR = 4.85) and respiratory diseases (SMR = 3.56) were predominant, with peak non-cancer mortality at 1–5 years (SMR = 2.33). Multivariate analysis identified advanced age, male sex, advanced-stage cancer, and lack of active treatment as key risk factors. This mortality shift, driven by aging and treatment-related complications, underscores the need for multidisciplinary survivorship programs. Prioritizing cardiovascular monitoring, lifestyle interventions (e.g., smoking cessation), and structured follow-up for untreated patients may reduce preventable deaths. These strategies address critical gaps in long-term management, improving survival and quality of life for lung cancer survivors.

**Supplementary Information:**

The online version contains supplementary material available at https://doi.org/10.1007/s13193-025-02458-7.

## Introduction

Lung cancer represents one of the most prevalent and lethal forms of malignant tumors on a global scale.Current estimates indicate that there are over 2 million new cases annually worldwide,resulting in approximately 1.8 million fatalities [1].In the United States,lung cancer remains the predominant cause of mortality among malignant neoplasms,with an estimated 230,000 new diagnoses and over 150,000 fatalities each year [2, 3].While advancements in targeted therapies and immunotherapy have improved survival rates, emerging evidence highlights non-cancer-related deaths as a critical determinant of long-term outcomes [4].Research indicates that non-cancer-related factors,particularly cardiovascular and respiratory diseases,have become principal contributors to mortality in lung cancer patients [5].Nonetheless, comprehensive analyses of temporal trends and modifiable risk factors associated with these deaths are limited, especially within large population-based cohorts.

The existing body of literature primarily concentrates on cancer-specific mortality, often neglecting the temporal dynamics of non-cancer-related mortality risks. Furthermore, current research is frequently limited by small sample sizes, brief follow-up durations, and insufficient adjustment for competing risks. To address these limitations, this study utilizes the SEER database (2000–2019) to:

1. SMRs for non-cancer-related deaths across distinct post-diagnosis intervals (< 1 year, 1–5 years, > 5 years);
2. Identify high-risk subgroups using Fine-Gray competing risk models;
3. Propose evidence-based strategies for mitigating preventable non-cancer mortality.

This study seeks to inform the development of customized survivorship care protocols for high-risk populations by analyzing temporal trends and their underlying determinants.

## Methods

### Data Source and Study Population

Data were extracted from the SEER Research Plus 18 Registries, covering the period from 2000 to 2019, utilizing SEER*Stat software (version 8.3.9.2). The study population comprised patients who were pathologically diagnosed with lung cancer, as defined by ICD-O-3 codes 8000–8230 and 8243–8245, 8250–8500. Cases identified exclusively through autopsy or death certificates, those with a history of prior malignancies, or those lacking essential variables such as survival time and cause of death, were excluded from the analysis (n = 12,457, representing 3.2% of the initial cohort). Missing data were addressed using multiple imputation, conducted over 10 iterations, for variables with less than 20% missingness. Information pertaining to cancer status (e.g., active disease, remission) at the time of death is not uniformly available in the SEER database. Consequently, all deaths subsequent to the initial lung cancer diagnosis were incorporated into the analysis, with the cause of death categorized according to the ICD-10 codes documented in the database. The potential confounding impact of active cancer status on non-cancer mortality (e.g., infection in the context of advanced disease) was recognized and taken into account during the interpretation of the results, especially for causes such as infections.

### Variables and Classification

Non-cancer-related mortality was classified into six categories according to ICD-10 codes: cardiovascular (I00-I99), respiratory (J00-J99), infectious (A00-B99), digestive (K00-K93), diabetes (E10-E14), and other causes. The covariates considered in the analysis comprised age (stratified into < 60 and > 60 years), sex, race, cancer stage (as per the AJCC 7th edition), treatment modalities (including surgery, chemotherapy, and radiotherapy), and marital status. Supplementary Table 1 provides detailed definitions of these non-cancer-related causes of death, along with their corresponding ICD-10 codes.

### Statistical Analysis

Initially, SMRs were computed for all causes of death, including LC, other cancers, and non-cancer-related diseases. The anticipated mortality figures for SMR computation were obtained from the mortality rates of the general population in the United States. These figures were adjusted for age, sex, race, and calendar year, utilizing the expected rate function integrated within SEER*Stat. Subsequently, SMRs for non-cancer-related causes of death were determined for LC patients across various follow-up intervals (< 1 year, 1–5 years, and > 5 years) post-diagnosis, with the population distribution and mean age (± standard deviation) stratified by relevant variables. The SMR was defined as the ratio of observed to expected deaths. The study population comprised individuals diagnosed with LC in the United States between 2000 and 2019, as recorded in the SEER database. A competing risk analysis utilizing the Fine-Gray model was conducted to adjust for potential confounders, including age, sex, race, summary stage, year of diagnosis, histologic type, treatment modalities (surgery, chemotherapy, and radiation therapy), and marital status, in order to assess the risks associated with mortality from non-cancer-related diseases and LC.All analyses were conducted utilizing SEER*Stat software (version 8.3.9.2) and Microsoft Excel 2019 (Microsoft Corporation, Redmond, WA). Statistical significance was determined using two-sided tests, with a p-value of less than 0.05 considered statistically significant.

## Ethical Statement

This study utilized publicly available de-identified data and was exempt from institutional review board approval.

## Results

### Baseline Characteristics

**Fig. 1:**
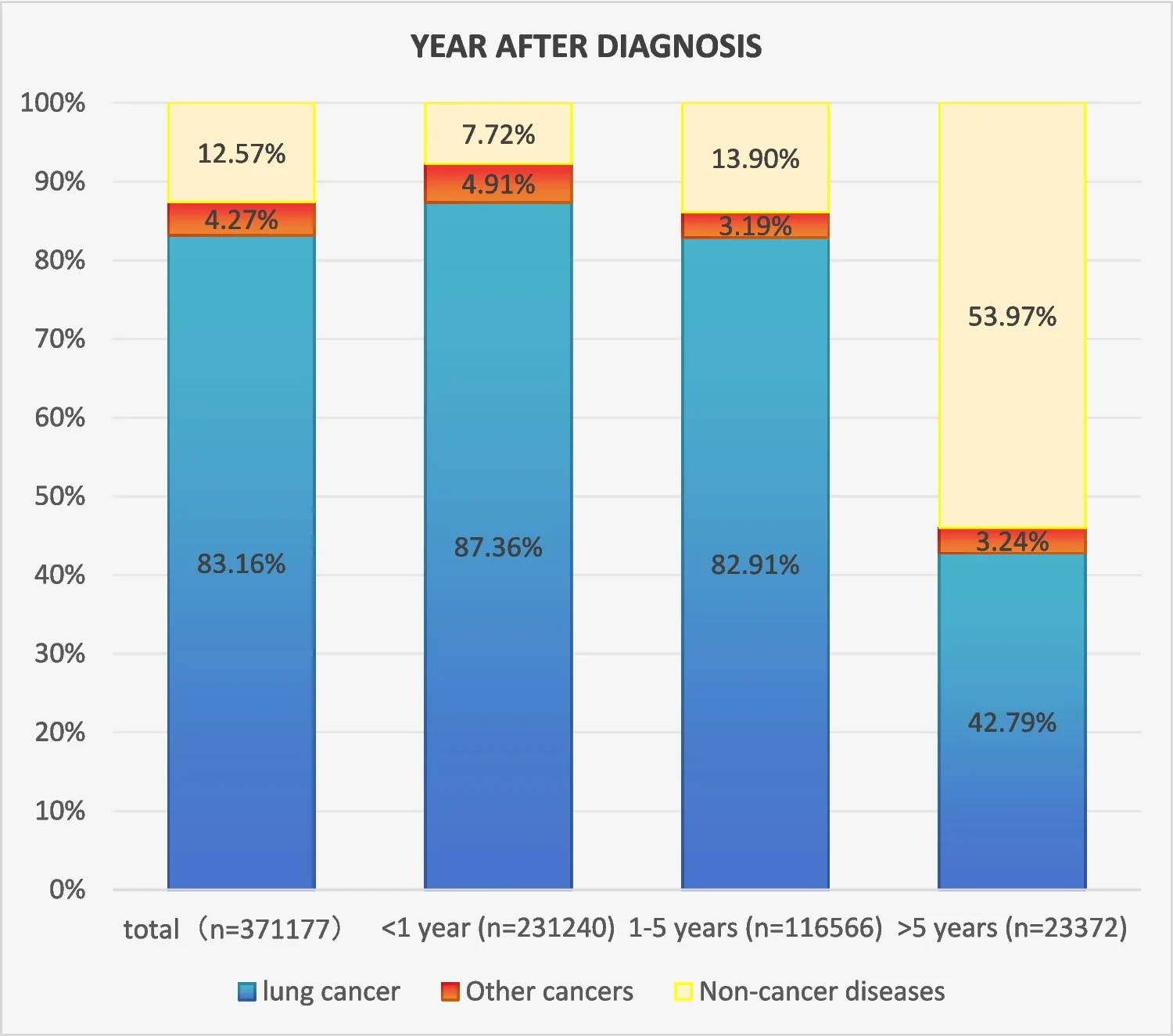
Percentage of all causes of death in lung cancer patients at different follow-up latency

Following an extensive analysis of the SEER database, a total of 371,177 eligible deaths were identified. Among the various causes of mortality in patients with LC, only the proportion of deaths due to non-cancer-related diseases exhibited an increase with prolonged follow-up time (Fig. 1), rising from 7.72% to 53.97%. Supplementary Table 2 presents the SMRs for all causes of death in LC patients post-diagnosis. The overall SMR was calculated to be 13.57 (95% CI [13.53–13.61]), with lung cancer exhibiting the highest SMR (173.96 [95% CI 173.35–174.58]), followed by other cancers (SMR 122.38 [95% CI 120.48–124.30]), and non-cancer-related diseases (SMR 2.24 [95% CI 2.22–2.26]).Among patients with LC, a total of 46,674 individuals succumbed to non-cancer-related diseases, with an average age at death of 70.49 ± 9.97 years. Of these individuals, 39,077 (83.72%) were over the age of 60 at the time of diagnosis, and 39,682 (85.02%) were identified as white. The highest proportion of deaths occurred within the first year following diagnosis (38.26%), followed by deaths occurring between one to five years post-diagnosis (34.71%). Table 1 presents selected baseline characteristics of LC patients who experienced non-cancer-related mortality, highlighting key trends across follow-up periods. Figure 1 illustrates the distribution of various causes of death among LC patients across different follow-up intervals.

**Table 1:** Baseline characteristics of lung cancer patients with non-cancer causes of death

Factors	overall		< 1 year		1–5 years		> 5 years	
	No	Age(mean ± SD)	No	Age(mean ± SD)	No	Age(mean ± SD)	No	Age(mean ± SD)
Total	46,674	70.49 ± 9.97	17,857	70.74 ± 10.19	16,202	70.89 ± 9.93	12,615	69.62 ± 9.64
Age(years)								
0–60 years	7597	54.21 ± 5.43	2897	54.12 ± 5.42	2481	54.32 ± 5.40	2219	54.22 ± 5.47
> 60 years	39,077	73.65 ± 7.17	14,960	73.96 ± 7.38	13,721	73.89 ± 7.24	10,396	72.90 ± 6.71
Sex								
Male	25,561	69.92 ± 9.88	10,764	70.30 ± 10.11	8,806	70.32 ± 9.71	5,991	68.66 ± 9.62
Female	21,113	71.18 ± 10.03	7,093	71.41 ± 10.29	7,396	71.57 ± 10.14	6,624	70.48 ± 9.58
Race								
White	39,682	70.87 ± 9.79	14,695	71.28 ± 9.92	13,803	71.28 ± 9.76	11,184	69.83 ± 9.58
Black	4,743	66.59 ± 10.65	2,238	66.62 ± 11.02	1,606	66.93 ± 10.47	899	65.9 ± 10.02
American Indian/Alaska Native	162	70.16 ± 9.30	70	70.19 ± 10.39	52	71.04 ± 7.63	40	68.98 ± 9.34
Asian or Pacific Islander	2,087	72.17 ± 9.76	854	72.37 ± 10.00	741	72.35 ± 10.05	492	71.58 ± 8.83
Summary stagc								
In situ								
Localized	17,542	71.94 ± 9.38	3,488	73.05 ± 9.20	7,129	72.56 ± 9.43	6,925	70.75 ± 9.29
Regional	14,948	69.93 ± 9.78	4,775	71.10 ± 9.59	5,640	70.12 ± 9.79	4,533	68.48 ± 9.77
Distant	14,184	69.27 ± 10.63	9,594	69.72 ± 10.67	3,433	68.7 ± 10.56	1,157	67.24 ± 10.23
Year of diagnosis								
2000–2004	13,769	69.53 ± 9.93	4,613	70.40 ± 10.21	3,760	70.16 ± 9.80	5,396	68.35 ± 9.66
2005–2009	14,055	70.31 ± 10.01	5,093	70.66 ± 10.41	4,357	70.58 ± 10.03	4,605	69.71 ± 9.54
2010–2014	12,434	71.08 ± 10	4,927	71.01 ± 10.24	4,988	71.22 ± 9.98	2,519	70.98 ± 9.60
2015–2019	6,416	71.13 ± 9.81	3,224	70.81 ± 9.86	3,097	71.36 ± 9.82	95	72.1 ± 9.06
Histologic type								
9.4.1 Small cell carcinoma—neuroendocrine carcinoma (NEC)	4,334	68.27 ± 9.68	2,549	69.34 ± 9.66	1,081	67.87 ± 9.54	704	65.04 ± 9.19
9.4.2 Non-small cell carcinoma	42,340	70.72 ± 9.97	15,308	70.97 ± 10.26	15,121	71.11 ± 9.92	11,911	69.89 ± 9.60
Surgery								
No	25,263	70.81 ± 10.38	14,197	70.77 ± 10.43	8,473	71.34 ± 10.25	2,891	69.41 ± 10.35
yes	21,411	70.11 ± 9.45	3,660	70.61 ± 9.25	7,729	70.42 ± 9.54	9,724	69.67 ± 9.43
Radiation therapy								
No	30,908	70.94 ± 9.74	12,010	71.41 ± 10.04	9,363	71.14 ± 9.67	9,535	70.17 ± 9.36
yes	15,766	69.63 ± 10.34	5,847	69.45 ± 10.36	6,839	70.56 ± 10.24	3,080	67.92 ± 10.28
Chemotherapy								
No/unknown	32,494	71.94 ± 9.73	12,401	72.10 ± 10.05	10,675	72.71 ± 9.57	9,418	70.85 ± 9.37
Yes	14,180	67.17 ± 9.72	5,456	67.65 ± 9.82	5,527	67.38 ± 9.66	3,197	65.98 ± 9.54
Marital status								
Single (never married)	5,646	65.15 ± 11.02	2,602	65.29 ± 11.15	1,846	65.33 ± 10.85	1,198	64.57 ± 10.97
Married (including common law)	23,073	70.37 ± 9.35	8,249	70.77 ± 9.47	7,993	70.78 ± 9.31	6,831	69.42 ± 9.16
Separated	488	65.74 ± 10.20	200	65.59 ± 10.61	187	65.53 ± 10.14	101	66.44 ± 9.53
Divorced	5,690	67.25 ± 9.46	2,204	67.74 ± 9.39	1,981	67.45 ± 9.59	1,505	66.29 ± 9.34
Widowed	9,870	76.13 ± 7.91	3,793	76.58 ± 8.15	3,493	76.56 ± 7.79	2,584	74.90 ± 7.60
Unmarried or Domestic Partner	48	66.40 ± 9.36	16	68.00 ± 7.79	23	65.96 ± 10.46	9	64.67 ± 9.50
Unknown	1,859	69.45 ± 10.53	793	70.13 ± 10.68	679	69.91 ± 10.21	387	67.22 ± 10.5

### SMR after LC Diagnosis

**Fig. 2:**
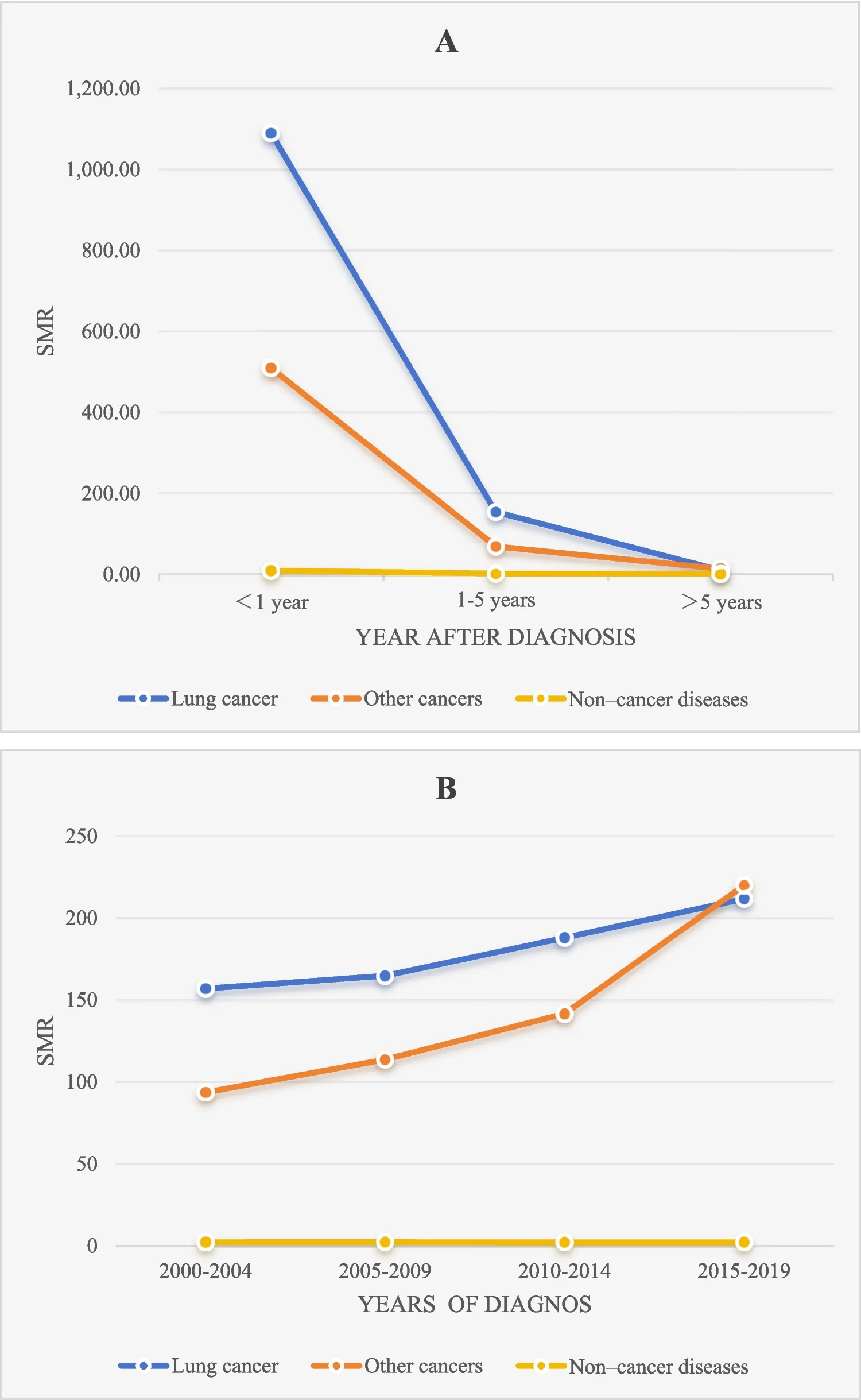
Trends in standardized mortality ratios for all causes of death in patients with lung cancer at different follow-up latency **A** and different year of diagnosis **B**

Figure 2A depicts a decrease in the SMR for all causes of death as the follow-up period extends. During the initial five years, LC presents the highest SMR, succeeded by other cancers and non-cancer-related diseases. Conversely, Fig. 2B illustrates an increasing trend in SMR for all causes associated with more recent years of diagnosis, with lung cancer consistently exhibiting the highest SMR levels.

**Fig. 3:**
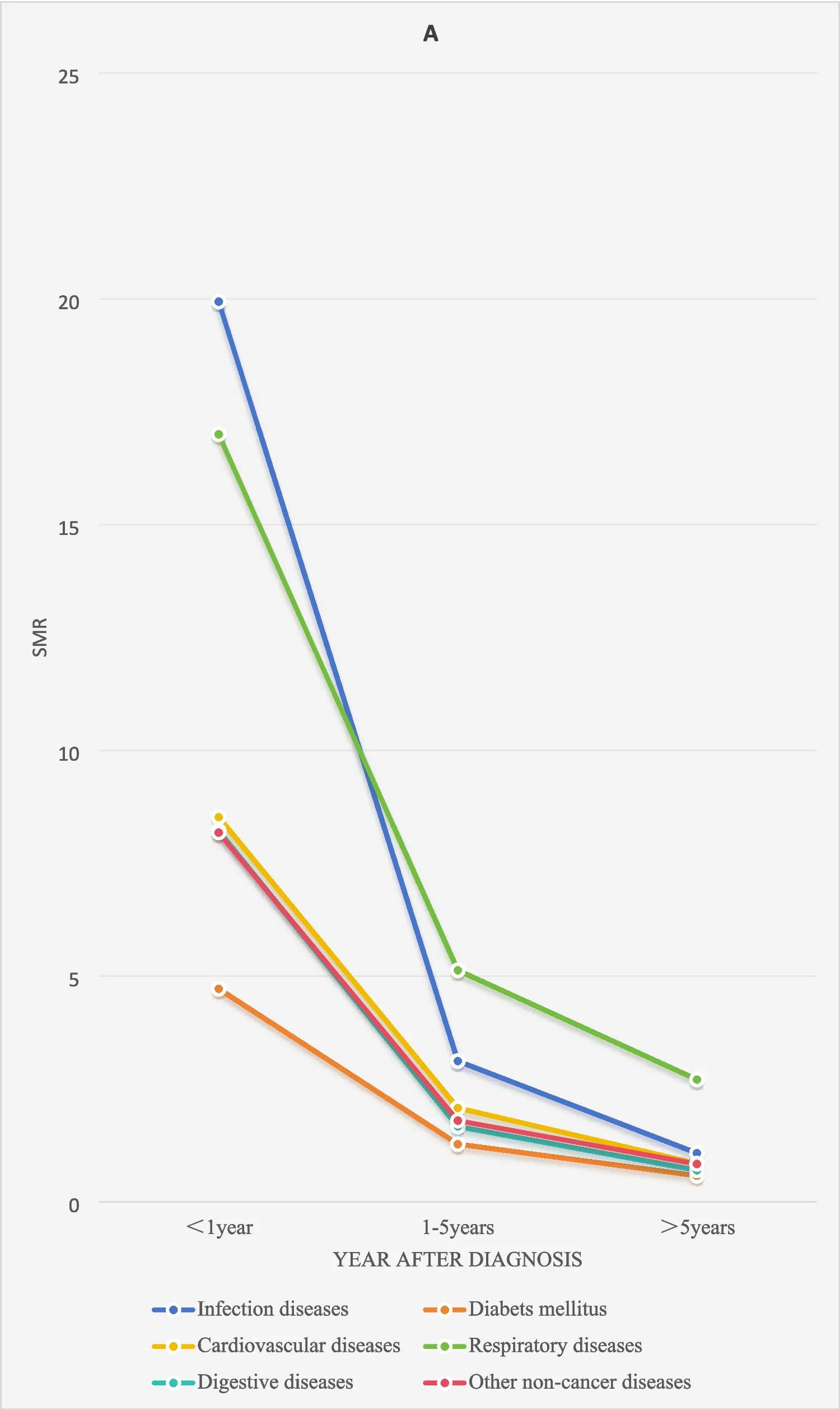

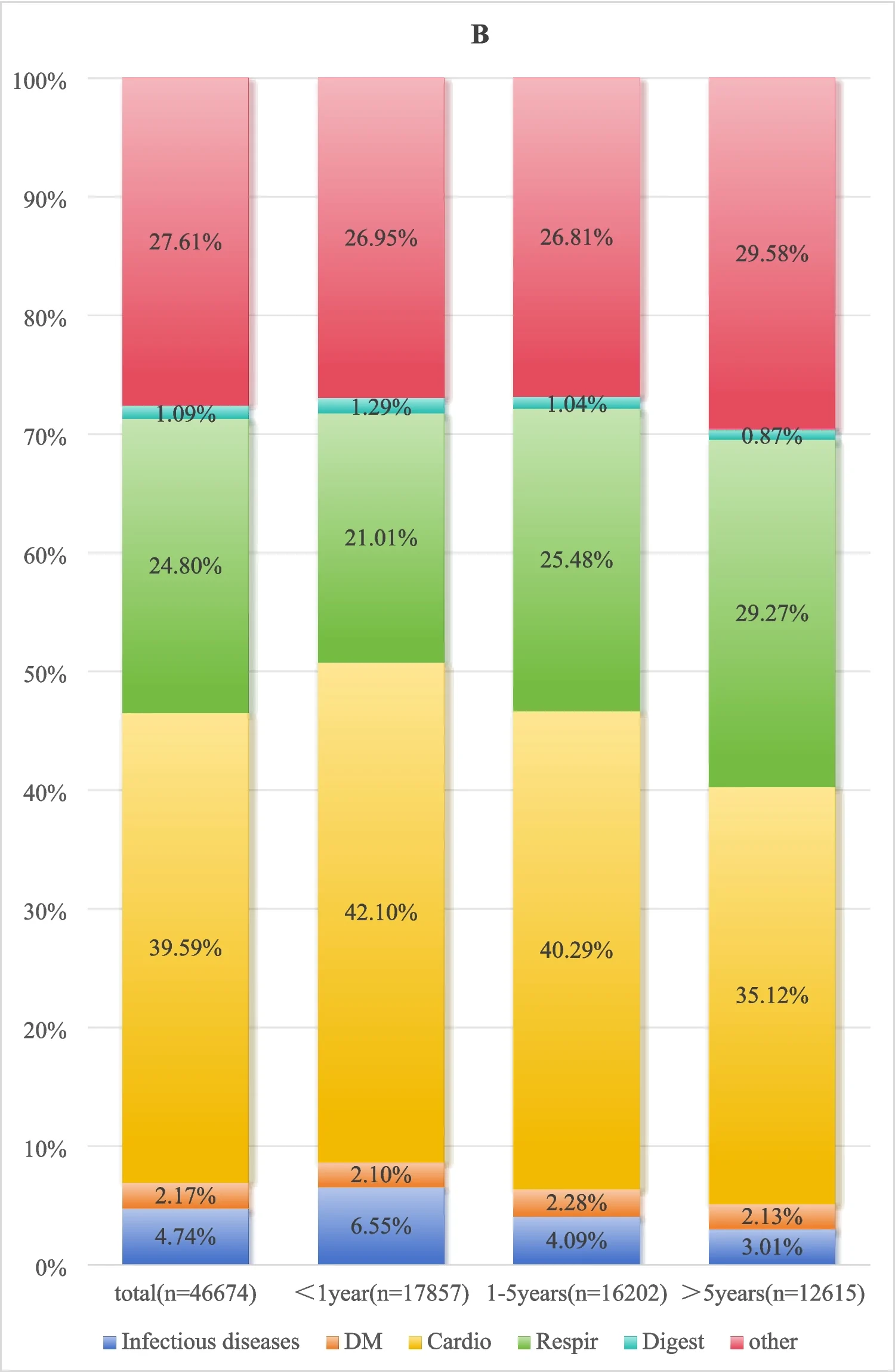
Trends in standardized mortality ratios **A** and proportions **B** of all non-cancer-related causes of death for patients with lung cancer at different follow-up periods after diagnosis

Figure 3A illustrates a decline in the SMR for six non-cancer-related diseases as the follow-up duration increased. Initially, infectious diseases exhibited the highest SMR within the first year. In subsequent follow-up periods, respiratory diseases demonstrated the highest SMR, followed by infectious diseases, whereas diabetes persistently displayed the lowest SMR. Figure 3B presents the proportional distribution of the six non-cancer-related diseases across different follow-up intervals, with cardiovascular diseases consistently comprising the largest proportion.

### Non‑Cancer Causes of Death at Different Follow‑Up Period

During the follow-up period, 46,674 out of 371,177 patients (12.57%) succumbed to non-cancer-related causes at an average age of 70.49 years (SD = 9.97). The overall SMR was calculated to be 2.24, with a 95% confidence interval ranging from 2.22 to 2.26.

Table 2 delineates the SMR for non-cancer-related causes of death among LC patients post-diagnosis, revealing an overall SMR of 2.24 (95% confidence interval [CI] 2.22–2.26). Notably, the SMR was consistently elevated within the first year following diagnosis. Table 3 illustrates the SMR for all non-cancer-related deaths across six physiological systems in LC patients after diagnosis, with the highest SMR recorded for respiratory diseases (SMR [95% CI] 4.85 [4.76–4.94]), followed by infectious diseases (SMR [95% CI] 3.56 [3.41–3.71]). (Note: Tables have been simplified in the supplementary material to highlight key trends).

**Table 2:** SMR of non-cancer causes of death after LC diagnosis

Factors	overall		＜1year		1-5years		＞5years	
	observed	SMR[95%CI]	observed	SMR[95%CI]	observed	SMR[95%CI]	observed	SMR[95%CI]
Total	46,674	2.24*[2.22,2.26]	17,857	9.61* [9.47, 9.76]	16,202	2.33* [2.29, 2.37]	12,615	1.05* [1.03, 1.07]
Age(years)								
0-60years	7597	4.55*[4.45,4.66]	2,897	26.47*[25.51, 27.45]	2,481	6.02*[5.79, 6.27]	2,219	1.93*[1.85, 2.02]
＞60years	39077	2.04*[2.02,2.06]	14,960	8.56*[8.42, 8.70]	13,721	2.10*[2.06, 2.13]	10,396	0.96*[0.94, 0.97]
Sex								
Male	25,561	2.48*[2.45,2.51]	10,764	9.42*[9.24, 9.60]	8,806	2.36*[2.31, 2.41]	5,991	1.10*[1.07, 1.13]
Female	21,113	2.01*[1.98,2.03]	7,093	9.92*[9.69, 10.15]	7,396	2.29*[2.24, 2.35]	6,624	1.01[0.98, 1.03]
Race								
White	39,682	2.21*[2.19,2.23]	14,695	9.36*[9.21, 9.51]	13,803	2.34*[2.30, 2.38]	11,184	1.07*[1.05, 1.09]
Black	4,743	2.62*[2.54,2.69]	2,238	10.92*[10.47, 11.38]	1,606	2.35*[2.24, 2.47]	899	0.97[0.91, 1.04]
American Indian/Alaska Native	162	4.35*[3.71,5.08]	70	17.32*[13.51, 21.89]	52	3.48*[2.60, 4.56]	40	2.19*[1.57, 2.99]
Asian or Pacific Islander	2,087	2.04*[1.95,2.13]	854	10.97*[10.24, 11.73]	741	2.05*[1.90, 2.20]	492	0.84*[0.77, 0.92]
Summary stagc								
In situ								
Localized	17,542	1.84*[1.81,1.87]	3,488	17.65*[17.07, 18.24]	7,129	2.89*[2.82, 2.95]	6,925	1.01[0.98, 1.03]
Regional	14,948	2.20*[2.16,2.23]	4,775	10.66*[10.36, 10.97]	5,640	2.41*[2.34, 2.47]	4,533	1.13*[1.10, 1.16]
Distant	14,184	3.16*[3.11,3.21]	9,594	7.91*[7.76, 8.07]	3,433	1.60*[1.55, 1.66]	1,157	1.02[0.96, 1.08]
Year of diagnosis								
2000-2004	13,769	2.25*[2.21,2.28]	4,613	8.44*[8.20, 8.69]	3,760	2.51*[2.43, 2.59]	5,396	1.32*[1.29, 1.36]
2005-2009	14,055	2.28*[2.25,2.32]	5,093	9.92*[9.65, 10.20]	4,357	2.70*[2.62, 2.78]	4,605	1.14*[1.11, 1.18]
2010-2014	12,434	2.21*[2.17,2.25]	4,927	9.77*[9.50, 10.05]	4,988	2.87*[2.79, 2.95]	2,519	0.74*[0.71, 0.77]
2015-2019	6,416	2.20*[2.15,2.25]	3,224	10.98*[10.61, 11.37]	3,097	1.47*[1.42, 1.52]	95	0.18*[0.15, 0.22]
Histologic type								
9.4.1 Small cell carcinoma - neuroendocrine carcinoma (NEC)	4,334	3.82*[3.70,3.93]	2,549	8.76*[8.42, 9.10]	1,081	2.22*[2.09, 2.36]	704	1.97*[1.83, 2.12]
9.4.2 Non-small cell carcinoma	42,340	2.15*[2.13,2.17]	15,308	9.77*[9.62, 9.93]	15,121	2.34*[2.30, 2.38]	11,911	1.02*[1.00, 1.04]
Surgery								
No	25,263	2.96*[2.92,3.00]	14,050	8.43*[8.29,8.57]	8,381	1.91*[1.87,1.96]	2,832	1.14*[1.09,1.18]
yes	21,411	1.74*[1.72,1.76]	3,807	19.95*[19.32, 20.59]	7,821	3.03*[2.97,3.10]	9,783	1.03[1.01,1.05]
Radiation therapy								
No	30,908	2.10*[2.08,2.13]	12,173	11.76*[11.55, 11.97]	9,510	2.36*[2.31, 2.41]	9,598	0.98[0.96, 1.00]
yes	15,766	2.57*[2.53,2.61]	5684	6.91*[6.73, 7.09]	6,692	2.28*[2.23, 2.34]	3,017	1.33*[1.28, 1.38]
Chemotherapy								
No/unknown	32,494	2.22*[2.19,2.24]	12,401	11.93*[11.72, 12.14]	10,675	2.54*[2.49, 2.59]	9,418	1.00[0.98, 1.02]
Yes	14,180	2.30*[2.26,2.33]	5,456	6.67*[6.49, 6.85]	5,527	2.01*[1.96, 2.06]	3,197	1.23*[1.18, 1.27]
Marital status								
Single (never married)	5,646	3.30*[3.22,3.39]	2,602	15.17*[14.59, 15.76]	1,846	3.11*[2.97, 3.26]	1,198	1.27*[1.20, 1.34]
Married (including common law)	23,073	2.07*[2.05,2.10]	8,249	9.11*[8.91, 9.30]	7,993	2.28*[2.23, 2.33]	6,831	1.02[0.99,1.04]
Separated	488	3.23*[2.95,3.53]	200	13.20*[11.44, 15.17]	187	3.78*[3.26, 4.37]	101	1.17[0.95, 1.42]
Divorced	5,690	3.13*[3.05,3.22]	2,204	14.26*[13.67, 14.87]	1,981	3.30*[3.16, 3.45]	1,505	1.42*[1.35, 1.49]
Widowed	9,870	1.86*[1.82,1.90]	3,793	7.04*[6.82, 7.27]	3,493	1.80*[1.74, 1.86]	2,584	0.91*[0.88, 0.95]
Unmarried or Domestic Partner	48	2.57*[1.89,3.41]	16	11.70*[6.69, 19.00]	23	1.90*[1.20, 2.85]	9	1.73[0.79, 3.28]
Unknown	1,859	2.62*[2.50,2.74]	793	11.32*[10.54, 12.13]	679	2.63*[2.43, 2.83]	387	1.01[0.92, 1.12]

**Table 3:** Non-cancer deaths in six systems

Non-cancer-related diseases	overall		< 1 year		1–5 years		> 5 years	
	observed	SMR[95%CI]	observed	SMR[95%CI]	observed	SMR[95%CI]	observed	SMR[95%CI]
Total	46,674	2.24* [2.22,2.26]	17,857	9.61* [9.47, 9.76]	16,202	2.33* [2.29, 2.37]	12,615	1.05* [1.03, 1.07]
Infection diseases	2,212	3.56* [3.41, 3.71]	1,169	19.94* [18.81, 21.11]	663	3.12* [2.89, 3.37]	380	1.08 [0.98, 1.20]
Diabets mellitus	1,014	1.22* [1.15, 1.30]	375	4.72* [4.25, 5.22]	370	1.28* [1.15, 1.42]	269	0.58* [0.51, 0.66]
Cardiovascular diseases	18,477	2.00* [1.97, 2.03]	7,518	8.52* [8.33, 8.72]	6,528	2.08* [2.03, 2.13]	4,431	0.85* [0.82, 0.87]
Respiratory diseases	11,573	4.85* [4.76, 4.94]	3,752	17.00* [16.46, 17.56]	4,128	5.13* [4.97, 5.29]	3,693	2.71* [2.62, 2.80]
Digestive diseases	509	1.78* [1.62, 1.94]	230	8.22* [7.19, 9.35]	169	1.67* [1.43, 1.94]	110	0.70* [0.57, 0.84]
Other non-cancer diseases	12,889	1.73* [1.70, 1.76]	4,813	8.18* [7.95, 8.41]	4,344	1.80* [1.75, 1.86]	3,732	0.84* [0.81, 0.86]

SMR, Standardized Mortality Ratio; CI, Confidence Interval. Bold text highlights the cause of death with the highest statistically significant SMR (95% CI lower limit > 1) within each follow-up period

### Within 5 Years after LC Diagnosis

17,857 patients (38.26%) succumbed within the first year, with a mean age of 70.74 ± 10.19 years. This cohort exhibited the highest overall SMR of 9.61 (95% confidence interval [CI]: 9.47, 9.76) (refer to Fig. 3B, Tables 1 and 2). Cardiovascular diseases emerged as the predominant non-cancer-related cause of mortality, with an SMR of 8.52 (95% CI: 8.33–8.72). Between one and five years post-diagnosis, 16,202 patients (34.71%) died from non-cancer-related causes, with a mean age of 70.89 ± 9.93 years and an overall SMR of 2.33 (95% CI: 2.29, 2.37) (see Fig. 3B, Tables 1 and 2). Cardiovascular diseases continued to be the leading non-cancer-related cause of death, with an SMR of 2.08 (95% CI: 2.03, 2.13). The mortality risk from non-cancer-related causes within five years post-LC diagnosis was significantly elevated compared to the general population, particularly concerning infectious and respiratory diseases.

### Over 5 Years after LC Diagnosis

12,615 patients, accounting for 27.03% of the cohort, succumbed to non-cancer-related diseases during the subsequent five-year follow-up period. The mean age at death was 69.62 years with a standard deviation of 9.64 years, and the overall SMR was calculated to be 1.05 (95% confidence interval [CI]: 1.03–1.07) (refer to Fig. 3B, Tables 1 and 2). Cardiovascular diseases were identified as the most prevalent non-cancer-related cause of mortality, with an SMR of 0.85 (95% CI: 0.82–0.87) (see Table 3). Notably, patients exhibited a significantly elevated risk of mortality from respiratory diseases, with an SMR of 2.71 (95% CI: 2.62–2.80), and from infectious diseases, with an SMR of 1.08 (95% CI: 0.98–1.20).

### Competing Risk Analysis

Multivariate competing risk models were employed to evaluate prognostic factors associated with LC-related mortality and mortality due to non-cancer-related causes among LC patients (Table 4.). Key findings from the competing risk analysis are summarized as follows: The analysis revealed that the risk of both types of mortality increased significantly with advancing age, with older patients demonstrating a higher likelihood of succumbing to non-cancer-related causes (Hazard Ratio [HR] [95% Confidence Interval (CI)] 1.857 [1.809, 1.907]). Female patients exhibited the lowest risk for both mortality types. Similarly, patients of Asian or Pacific Island descent were found to have the lowest risk for both outcomes. The presence of distant metastases was associated with a substantially elevated risk of LC-related death (HR [95% CI] 3.277 [3.229, 3.325]), while concurrently exhibiting a markedly reduced risk of death from non-cancer-related causes (HR [95% CI] 0.291 [0.283, 0.300]). Patients diagnosed between 2015 and 2019 demonstrated the lowest risk for both mortality types. Those with non-small cell carcinoma had the lowest risk of LC-related mortality. Conversely, patients who underwent surgery and radiation therapy were at the highest risk for mortality due to non-cancer-related causes. Chemotherapy was associated with the lowest risk for both mortality types. Widowed patients were identified as having the highest risk for both LC-related and non-cancer-related mortality.

**Table 4:** Multivariate competing risk analysis for non-cancer diseases and lung cancer-related hazard ratio

Factors	Cause of death			
	Lung cacer		Non-cacer diseases	
	HR[95%CI]	p	HR[95%CI]	p
Age(years)				
0-60years	Ref.		Ref.	
60+years	1.103[1.094,1.113]	＜0.05	1.857[1.809,1.907]	＜0.05
Sex				
Male	Ref.		Ref.	
Female	0.871[0.864,0.878]	＜0.05	0.818[0.802,0.834]	＜0.05
Race				
White	Ref.		Ref.	
Black	0.956[0.944,0.968]	＜0.05	1.031[0.998,1.065]	0.063
American Indian/Alaska Native	1.034[0.973,1.098]	0.278	0.916[0.780,1.075]	0.283
Asian or Pacific Islander	0.872[0.859,0.885]	＜0.05	0.718[0.686,0.752]	＜0.05
Summary stagc				
In situ				
Localized	Ref.		Ref.	
Regional	2.052[2.023,2.081]	＜0.05	0.656[0.641,0.672]	＜0.05
Distant	3.277[3.229,3.325]	＜0.05	0.291[0.283,0.300]	＜0.05
Year of diagnosis				
2000-2004	Ref.		Ref.	
2005-2009	0.883[0.874,0.893]	＜0.05	1.046[1.021,1.073]	＜0.05
2010-2014	0.804[0.795,0.813]	＜0.05	0.977[0.952,1.002]	0.074
2015-2019	0.662[0.654,0.670]	＜0.05	0.825[0.801,0.849]	＜0.05
Histologic type				
9.4.1 Small cell carcinoma - neuroendocrine carcinoma (NEC)	Ref.		Ref.	
9.4.2 Non-small cell carcinoma	0.900[0.891,0.910]	＜0.05	1.112[1.072,1.153]	＜0.05
Surgery				
No	Ref.		Ref.	
yes	0.429[0.424,0.434]	＜0.05	1.473[1.437,1.509]	＜0.05
Radiation therapy				
No	Ref.		Ref.	
yes	1.024[1.015,1.032]	＜0.05	1.089[1.064,1.114]	＜0.05
Chemotherapy				
No/unknown	Ref.		Ref.	
Yes	0.805[0.797,0.812]	＜0.05	0.736[0.720,0.753]	＜0.05
Marital status				
Single (never married)	Ref.		Ref.	
Married (including common law)	0.973[0.961,0.985]	＜0.05	0.895[0.867,0.923]	＜0.05
Separated	1.014[0.978,1.052]	0.447	1.017[0.924,1.120]	0.726
Divorced	1.033[1.017,1.050]	＜0.05	1.034[0.995,1.075]	0.087
Widowed	1.043[1.027,1.059]	＜0.05	1.136[1.096,1.177]	＜0.05
Unmarried or Domestic Partner	1.072[0.961,1.195]	0.212	0.826[0.612,1.116]	0.213
Unknown	0.993[0.970,1.016]	0.527	1.022[0.967,1.080]	0.432

## Discussion

Despite continuous advancements in lung cancer treatments, including chemotherapy, radiotherapy, surgery, and targeted therapies [6, 7], our large population-based study reveals that non-cancer-related mortality constitutes a major, and oftenoverlooked, challenge to overall survival.Our study, employing a large population-based cohort, identifies a significant burden of non-cancer-related mortality among lung cancer patients, characterized by distinct temporal patterns and risk profiles. The principal findings are as follows:

1. Non-cancer-related deaths represent a substantial and increasing proportion of overall mortality over time, surpassing cancer-specific deaths beyond five years post-diagnosis;
2. The risk of non-cancer mortality is highest within the first year (SMR = 9.61) and remains elevated during the 1–5 year period (SMR = 2.33);
3. Cardiovascular and respiratory diseases are the predominant non-cancer causes of death;
4. High-risk subgroups include older adults, males, Caucasians, patients with advanced-stage disease, and those not receiving active treatment.

Beyond five years post-diagnosis, non-cancer deaths exceed cancer-specific ones,a trend seen in other cancer groups [8]. The increased risk within 1–5 years (SMR = 2.33) highlights the need for close monitoring due to treatment-related cardiotoxicity and immunosuppression [9, 10].

Cardiovascular diseases were the leading non-cancer cause of death (SMR = 4.85), supporting Sturgeon et al.'s findings [11]. This elevated risk likely stems from shared risk factors like smoking and the cumulative effects of treatments, such as radiotherapy-induced myocardial fibrosis [10] and anthracycline-induced cardiotoxicity [9].

Respiratory diseases (SMR = 4.85) and infectious diseases (SMR = 3.56) were significant contributors to mortality. This observation aligns with the notably high prevalence of respiratory symptoms, such as dyspnea, among lung cancer patients [12–14]. This prevalence is further exacerbated by the high incidence of pre-existing chronic obstructive pulmonary disease (COPD), which exceeds 50%[15],the direct impact of the tumor on pulmonary function, and pulmonary toxicitiesassociated with treatment. For example, bleomycin can cause severe lung toxicity, and immune checkpoint inhibitors are linked to a significant risk of pneumonitis [16, 17]. Furthermore, patients demonstrate an increased susceptibility.

to pathogens due to compromised immune function,potentially mediated by tumor-induced immunosuppressive pathways [18], thereby heightening the risk of respiratory and other infections.

Although our study identified a comparatively lower SMR for diabetes at 1.08,

the complexity of its role persists. Our findings align with those of Yang et al.,who identified no independent risk associated with diabetes in non-smokers [19],which contrasts with studies suggesting protective effects of metformin [20–22].

Our findings advocate for the establishment of multidisciplinary survivorship programs that incorporate the expertise of oncologists, cardiologists, and pulmonologists. Epidemiological evidence indicates that interventions focusing on lifestyle and environmental factors can significantly decrease cancer incidence [23, 24],highlighting their critical role in survivorship care. Essential strategies should encompass:

1. **Risk-adapted treatment:** De-escalation of therapy in early-stage patients to reduce long-term toxicity [25].
2. **Comorbidity management:** Aggressive management of cardiovascular and respiratory comorbidities is essential, incorporating the administration of statins for cardiovascular prevention [11], the use of prophylactic antibiotics, and the implementation of pulmonary rehabilitation for patients with COPD[15, 26].
3. **Personalized survivorship care:** High-risk subgroups (e.g., elderly males, patients with advanced disease) require customized care plans, incorporating regular cardiopulmonary function evaluations (e.g., annual echocardiography[10]) into follow-up protocols.
4. **Patient empowerment:** Utilization of mobile health tools for symptom tracking and promoting healthy lifestyles (smoking cessation, physical activity)[27].

## Limitations

This study has several limitations. Firstly, the SEER database lacks detailed comorbidity information, such as smoking pack-years and lipid profiles, which may introduce confounding in the SMR estimates. Moreover, the absence of data regarding cancer status (active or in remission) at the time of death could confound the observed associations between certain non-cancer causes, such as infections, and mortality. Secondly, the analysis did not encompass detailed treatment information, including specific chemotherapy cycles. Future research should integrate electronic health records to more accurately capture real-world treatment toxicity and comorbidity profiles [22]. Despite these limitations, the applicationof competing risk analysis and the large sample size enhance the robustness ofour primary findings.

## Conclusion

The variety of causes of mortality among lung cancer patients and the progression of non-cancer-related mortality risk over time demonstrate a clear temporal trend in causes of death at various intervals following diagnosis. Following diagnosis, the causes of mortality among lung cancer patients exhibit a distinct temporal trend. Lung cancer remains the predominant cause of death within the first year post-diagnosis; however, over time, the influence of other malignancies and non-cancer-related diseases progressively increases. Specifically, the incidence of non-cancer-related diseases demonstrated a significant increase over the period spanning 1 to 5 years and extending beyond 1 year post-diagnosis, exceeding the incidence of lung cancer itself. Critical determinants influencing non-cancer-related mortality include age, gender, ethnicity, cancer stage, and treatment modality. Older adults, males, individuals of Caucasian ethnicity, patients with advanced-stage cancer, and those not undergoing active treatment are at heightened risk.This study, for the first time, has systematically examined the standardized mortality ratio for non-cancer-related deaths in lung cancer patients and identified the key factors influencing these risks. These findings provide crucial evidence for developing long-term, comprehensive management strategies for lung cancer patients.

## Supplementary Information

Below is the link to the electronic supplementary material.

ESM 1 (DOCX 15.5 KB)

ESM 2 (DOCX 22.7 KB)

## Data Availability

The datasets generated and/or analyzed during the current study are available in the Surveillance, Epidemiology, and End Results (SEER) database, which is publicly accessible at https://seer.cancer.gov/. The specific data used in this study can be accessed through the SEER*Stat software, version 8.3.9.2, using the following submission: Incidence-SEER Research Plus Data, 18 Registries (excluding Alaska), November 2020 Submission. For further details regarding data access and usage, please refer to the SEER database guidelines and policies.
