# Supplementary material for: Non-Cancer-Related Causes' Impact on Lung Cancer Survival: SEER Analysis
Source: Indian J Surg Oncol. 2025 Oct 24;17(7):1542–54. doi: 10.1007/s13193-025-02458-7 (PMC13437860; doi:10.1007/s13193-025-02458-7)
Supplement: Supplementary file 2 [file 13193_2025_2458_MOESM2_ESM.docx]

| Supplementary Table2 SMR of all causes of death after LC diagnosis | | | | | | | | |
| --- | --- | --- | --- | --- | --- | --- | --- | --- |
| Factors | Overall | | Causes of death | | | | | |
|  |  |  | Lung cancer | | Other cancers | | Non–cancer diseases | |
|  | Observed | SMR [95% CI] | Observed | SMR [95% CI] | Observed | SMR [95% CI] | Observed | SMR [95% CI] |
| Total | 371,177 | 13.57*[13.53, 13.61] | 308,661 | 173.96*[173.35,174.58] | 15,842 | 122.38*[120.48, 124.30] | 46,674 | 2.24*[2.22,2.26] |
| Age(years) |  |  |  |  |  |  |  |  |
| 0-60years | 94,432 | 38.39*[38.14,38.63] | 82,109 | 380.09*[377.50,382.70] | 4,726 | 329.39*[320.07, 338.92] | 7,597 | 4.55*[4.45,4.66] |
| ＞60years | 276,745 | 11.12*[11.08, 11.16] | 226,552 | 145.39*[144.79,145.99] | 11,116 | 96.57*[94.78, 98.38] | 39,077 | 2.04*[2.02,2.06] |
| Sex |  |  |  |  |  |  |  |  |
| Male | 205,567 | 14.80*[14.74, 14,86] | 171,389 | 169.48*[168.67,170.28] | 8,617 | 118.74*[116.25, 121.28] | 25,561 | 2.48*[2.45,2.51] |
| Female | 165,610 | 12.30*[12.24, 12,36] | 137,272 | 179.91*[178.96,180.87] | 7,225 | 127.01*[124.10, 129.97] | 21,113 | 2.01*[1.98,2.03] |
| Race |  |  |  |  |  |  |  |  |
| White | 306,878 | 13.04*[12.99,13.08] | 254,359 | 165.63*[164.98, 166.27] | 12,837 | 118.21*[116.17, 120.27] | 39,682 | 2.21*[2.19,2.23] |
| Black | 40,595 | 16.79*[16.62,16.95] | 33,781 | 214.75*[212.47, 217.06] | 2,071 | 132.26*[126.63, 138.09] | 4,743 | 2.62*[2.54,2.69] |
| AIAK | 1,473 | 29.46*[27.97, 31.00] | 1,247 | 405.09*[382.92, 428.22] | 64 | 169.33*[130.40, 216.23] | 162 | 4.35*[3.71,5.08] |
| API | 22,231 | 16.53*[16.31, 16.74] | 19,274 | 246.57*[243.10, 250.07] | 870 | 180.49*[168.70, 192.89] | 2,087 | 2.04*[1.95,2.13] |
| Summary stage |  |  |  |  |  |  |  |  |
| Localized | 45,574 | 3.70*[3.67, 3.74] | 26,895 | 36.87*[36.43, 37.31] | 1,137 | 28.46*[26.83, 30.17] | 17,542 | 1.84*[1.81,1.87] |
| Regional | 88,393 | 9.82*[9.75, 9.88] | 71,114 | 116.97*[116.11, 117.83] | 2,331 | 59.13*[56.76, 61.58] | 14,948 | 2.20*[2.16,2.23] |
| Distant | 237,210 | 39.23*[39.07,39.38] | 210,652 | 482.17*[480.12, 484.24] | 12,374 | 247.05*[242.72, 251.45] | 14,184 | 3.16*[3.11,3.21] |
| Year of diagnosis |  |  |  |  |  |  |  |  |
| 2000-2004 | 107,382 | 13.24*[13.16, 13.32] | 89,032 | 157.00*[155.97, 158.03] | 4,581 | 93.62*[90.93, 96.37] | 13,769 | 2.25*[2.21,2.28] |
| 2005-2009 | 106,926 | 13.21*[13.13, 13.29] | 88,463 | 164.78*[163.69, 165.87] | 4,408 | 113.66*[110.33, 117.07] | 14,055 | 2.28*[2.25,2.32] |
| 2010-2014 | 102,140 | 13.87*[13.79, 13.96] | 85,493 | 188.01*[186.75, 189.27] | 4,213 | 141.66*[137.42, 146.01] | 12,434 | 2.21*[2.17,2.25] |
| 2015-2019 | 54,729 | 14.45*[14.33, 14.58] | 45,673 | 211.84*[209.90, 213.79] | 2,640 | 220.01*[211.70, 228.57] | 6,416 | 2.20*[2.15,2.25] |
| Histologic type |  |  |  |  |  |  |  |  |
| SCLC | 61,507 | 39.11*[38.80, 39.42] | 54,483 | 428.61*[425.02, 432.23] | 2,690 | 241.80*[232.75, 251.12] | 4,334 | 3.82*[3.70,3.93] |
| NSCLC | 309,670 | 12.01*[11.97, 12.05] | 254,178 | 154.31*[153.71, 154.91] | 13,152 | 111.15*[109.26, 113.06] | 42,340 | 2.15*[2.13,2.17] |
| Surgery |  |  |  |  |  |  |  |  |
| No | 301,731 | 26.86*[26.76, 26.96] | 262,953 | 356.33*[354.97, 357.69] | 13,515 | 200.31*[196.95, 203.72] | 25,263 | 2.96*[2.92,3.00] |
| yes | 69,446 | 4.31*[4.28, 4.34] | 45,708 | 44.11*[43.07, 44.51] | 2,327 | 37.54*[36.03, 39.10] | 21,411 | 1.74*[1.72,1.76] |
| Radiation therapy |  |  |  |  |  |  |  |  |
| No | 206,994 | 10.70*[10.66, 10.75] | 166,021 | 136.09*[135.44, 136.75] | 9,692 | 110.92*[108.73, 113.15] | 31，281 | 2.11*[2.09,2.13] |
| yes | 164,183 | 20.48*[20.38, 20.58] | 142,640 | 257.30*[255.97, 258.64] | 6,150 | 146.16*[142.53, 149.86] | 15,393 | 2.56*[2.52,2.60] |
| Chemotherapy |  |  |  |  |  |  |  |  |
| No/unknown | 189,427 | 10.04*[9.99, 10.08] | 147,375 | 131.89*[131.22, 132.56] | 9,558 | 119.21*[116.84, 121.63] | 32,494 | 2.22*[2.19,2.24] |
| Yes | 181,750 | 21.43*[21.33, 21.52] | 161,286 | 245.54*[244.34, 246.74] | 6,284 | 127.52*[124.39, 130.71] | 14,180 | 2.30*[2.26,2.33] |
| Marital status |  |  |  |  |  |  |  |  |
| Single | 50,248 | 21.83*[21.64, 22.02] | 42,065 | 259.12*[256.65, 261.61] | 2,537 | 201.27*[193.52, 209.26] | 5,646 | 3.30*[3.22,3.39] |
| Married | 190,661 | 12.83*[12.77, 12.89] | 159,676 | 154.03*[153.28, 154.79] | 7,912 | 111.63*[109.18, 114.11] | 23,073 | 2.07*[2.05,2.10] |
| Separated | 4,439 | 21.50*[20.87, 22.14] | 3,755 | 245.12*[237.34, 253.09] | 196 | 147.84*[127.86, 170.04] | 488 | 3.23*[2.95,3.53] |
| Divorced | 45,794 | 18.64*[18.47, 18.81] | 38,158 | 212.73*[210.60, 214.88] | 1,946 | 148.82*[142.28, 155.58] | 5,690 | 3.13*[3.05,3.22] |
| Widowed | 65,270 | 9.94*[9.86, 10.01] | 52,786 | 166.13*[164.72, 167.55] | 2,614 | 94.34*[90.76, 98.03] | 9,870 | 1.86*[1.82,1.90] |
| Unmarried | 482 | 19.16*[17.48, 20.95] | 414 | 245.91*[222.79, 270.78] | 20 | 217.82*[133.05, 336.40] | 48 | 2.57*[1.89,3.41] |
| Unknown | 14,283 | 15.27*[15.02, 15.52] | 11,807 | 192.93*[189.46, 196.44] | 617 | 163.74*[151.08, 177.19] | 1,859 | 2.62*[2.50,2.74] |
